# Exploring the patterns in traditional Chinese medicine for bipolar disorder: a data-driven network approach

**DOI:** 10.3389/fphar.2025.1524345

**Published:** 2025-06-04

**Authors:** Zhenshan Sun, Jiangbangrui Chu, Junjie Peng, Kefan Hu, Zhengyi Wang, Zhu Zhang, Ken Kin Lam Yung

**Affiliations:** ^1^ Department of Biology, Faculty of Science, Hong Kong Baptist University, Kowloon, Hong Kong SAR, China; ^2^ Golden Meditech Center for NeuroRegeneration Sciences, Hong Kong Baptist University, Kowloon, Hong Kong SAR, China; ^3^ Teaching and Research Division, School of Chinese Medicine, Hong Kong Baptist University, Kowloon, Hong Kong SAR, China; ^4^ Hong Kong Baptist University, Kowloon, Hong Kong SAR, China; ^5^ Cuiying Honors College, Lanzhou University, Lanzhou, China; ^6^ Department of Science and Environmental Studies, The Education University of Hong Kong, Kowloon, China

**Keywords:** bioinformatics, data mining, bipolar disorder, Chinese medicine, network analysis

## Abstract

**Aim of the study:**

Traditional Chinese Medicine offers a holistic approach that could provide complementary benefits for bipolar disorder treatment. However, the clinical cases in Traditional Chinese Medicine are highly dispersed, creating challenges for translational research. This study employs a novel data-mining-derived approach to identify treatment patterns and active metabolite interactions within these clinical cases.

**Methods and materials:**

Bipolar disorder-related targets were determined using DisGeNET and GeneCards databases. Active botanical drugs were extracted from the BATMAN-TCM 2.0 database. All terms for botanical drugs and diseases were confirmed via the Pharmacopoeia of the People’s Republic of China 2020 Edition and Medical Subject Headings. Networks were constructed using Cytoscape, with data analysis performed using Python. MTT cell viability and qRT-PCR analysis were used to perform *in vitro* experiments on SH-SY5Y neuroblastoma cells.

**Results:**

Five key botanical drugs—*Glycyrrhizae Radix Et Rhizoma*, *Poria*, *Coptidis Rhizoma*, *Bupleuri Radix*, and *Polygalae Radix*—were identified as core drugs in BD treatment formulas. The botanical drug-metabolite-target network was constructed. *In vitro* experiments using SH-SY5Y neuroblastoma cells demonstrated dose-dependent effects of palmitic acid (PA) and stearic acid (SA) on cell viability and gene expression. qRT-PCR analysis revealed bidirectional regulation of GABRA1 and ESR1 by these metabolites.

**Conclusion:**

Five botanical drugs: *Glycyrrhizae Radix Et Rhizoma*, *Poria*, *Coptidis Rhizoma*, *Bupleuri Radix*, and *Polygalae Radix*, were identified as the core botanical drugs in bipolar disorder treatment. The main mechanism of these botanical drugs is their effects on the gamma-aminobutyric acid type A receptor and ESR1.

## 1 Introduction

Bipolar disorder (BD) is a chronic and recurrent affective psychiatric illness characterized by extreme mood fluctuations between mania and depression, which can severely impact an individual’s daily life and overall wellbeing (Smith et al., 2012). The number of individuals affected by BD increased by 59.3% from 1990 to 2019, exceeding 39 million (Lai et al., 2024). Moreover, birth cohort effects indicate a higher prevalence among younger populations (Lai et al., 2024), posing an increasingly serious challenge for the management of BD.

Current clinical solutions for BD include mood stabilizers (Lithium, valproate, etc.), antipsychotics (olanzapine, Quetiapine, etc.), antidepressants (paroxetine, citalopram, etc.), and psychotherapy. However, each of them has limitations. Valproate imposes a significant hepatic burden (Zhu et al., 2024), while lithium may lead to nephrotoxicity and suppression of thyroid function (Phelps and Coskey, 2024). Due to the need for close dosage monitoring and long-term use, patient adherence is often compromised (Chen et al., 2022). Antipsychotics are associated with metabolic disturbances, including weight gain and abnormalities in glucose and lipid levels, and may cause cognitive side effects (Solmi et al., 2024; Wong et al., 2024), with limited tolerability in elderly and pediatric populations. Antidepressants are only applicable during the depressive phase of bipolar disorder, and their efficacy remains controversial (Gottlieb and Young, 2024). Psychotherapy has limited effectiveness, typically requiring long-term and intensive intervention (Miklowitz et al., 2021). Clinical practice calls for the development of safe, long-acting, and mild adjunctive therapies that also support multidimensional functional regulation—an area where Traditional Chinese Medicine (TCM) shows considerable potential.

Traditional Chinese Medicine (TCM) presents a unique reservoir of knowledge and therapeutic practices developed over thousands of years (Dong, 2013). Its holistic view of health, which emphasizes the balance of body and mind, offers a promising alternative for addressing the complexities of bipolar disorder (Zhang et al., 1994). TCM attributes the onset of bipolar disorder to emotional dysregulation, disturbances in qi and blood, disruption of Yin and Yang balance, and dysfunction of the internal organs. It views mood fluctuations not merely as the result of isolated neurobiochemical abnormalities, but also as an external expression of an imbalance in body and mind (Chen et al., 2024). TCM offers a unique therapeutic approach characterized by multi-target actions (Lo et al., 2023), holistic regulation (Xi et al., 2025), and individualized treatment based on syndrome differentiation (Li et al., 2021). It can simultaneously modulate neurotransmission, endocrine function, and immune responses, with low side effects (Deng et al., 2022). These features confer complementary advantages that are difficult to replicate with conventional pharmacotherapy, particularly in improving emotional stability, enhancing treatment adherence, and reducing side effects. As such, TCM is especially suitable for maintenance-phase interventions and long-term mood stabilization. Hence, drawing on the clinical experience of TCM offers a promising avenue for informing and inspiring novel drug development strategies for bipolar disorder. However, several obstacles hinder the translation of TCM practices into effective clinical interventions.

One major challenge is the philosophical gap between TCM and Western medicine (Dong, 2013). TCM approaches mental health through a lens of systemic balance, which can lead to skepticism about its effectiveness, especially in the context of evidence-based medicine (Zhao et al., 2023). Additionally, the diverse methodologies inherent in TCM—including varying diagnostic criteria and treatment strategies—often result in inconsistent practices, complicating the establishment of standardized treatment protocols (Che et al., 2024).

Despite these challenges, there is a treasure trove of clinical experiences documented in TCM, particularly in the management of bipolar disorder through botanical drug formulas and case studies. However, the potential of these insights has not been fully realized, mainly due to a lack of systematic approaches for analyzing and integrating this data7 (Chen et al., 2024). This study aims to utilize bioinformatics, machine learning (ML), and network pharmacology to create a data-mining-derived approach (DMDA) that can effectively harness TCM knowledge for bipolar disorder treatment, combining experimental validation of screened targets to validate identified TCM therapeutic targets, paving the way for the development of novel therapeutic options.

By merging traditional wisdom with contemporary scientific techniques, this research aspires to bridge the gap between empirical insights and clinical application, ultimately fostering a new sight in drug development for bipolar disorder.

## 2 Materials and methods

### 2.1 Reagents

Dulbecco’s Modified Eagle Medium (DMEM) and fetal bovine serum (FBS) were purchased from Gibco (United States). Penicillin/streptomycin (P/S), dimethyl sulfoxide (DMSO), and MTT reagent were obtained from Sigma-Aldrich (United States). P Palmitic Acid (PA) and Stearic Acid (SA) were obtained from Chengdu Must Bio-technology Co., LTD. TRIzol and qRT-PCR reagents (reverse transcriptase, SYBR Green) were acquired from Thermo Fisher Scientific (United States).

### 2.2 Formulas & botanical drugs selection

**FIGURE 1:**
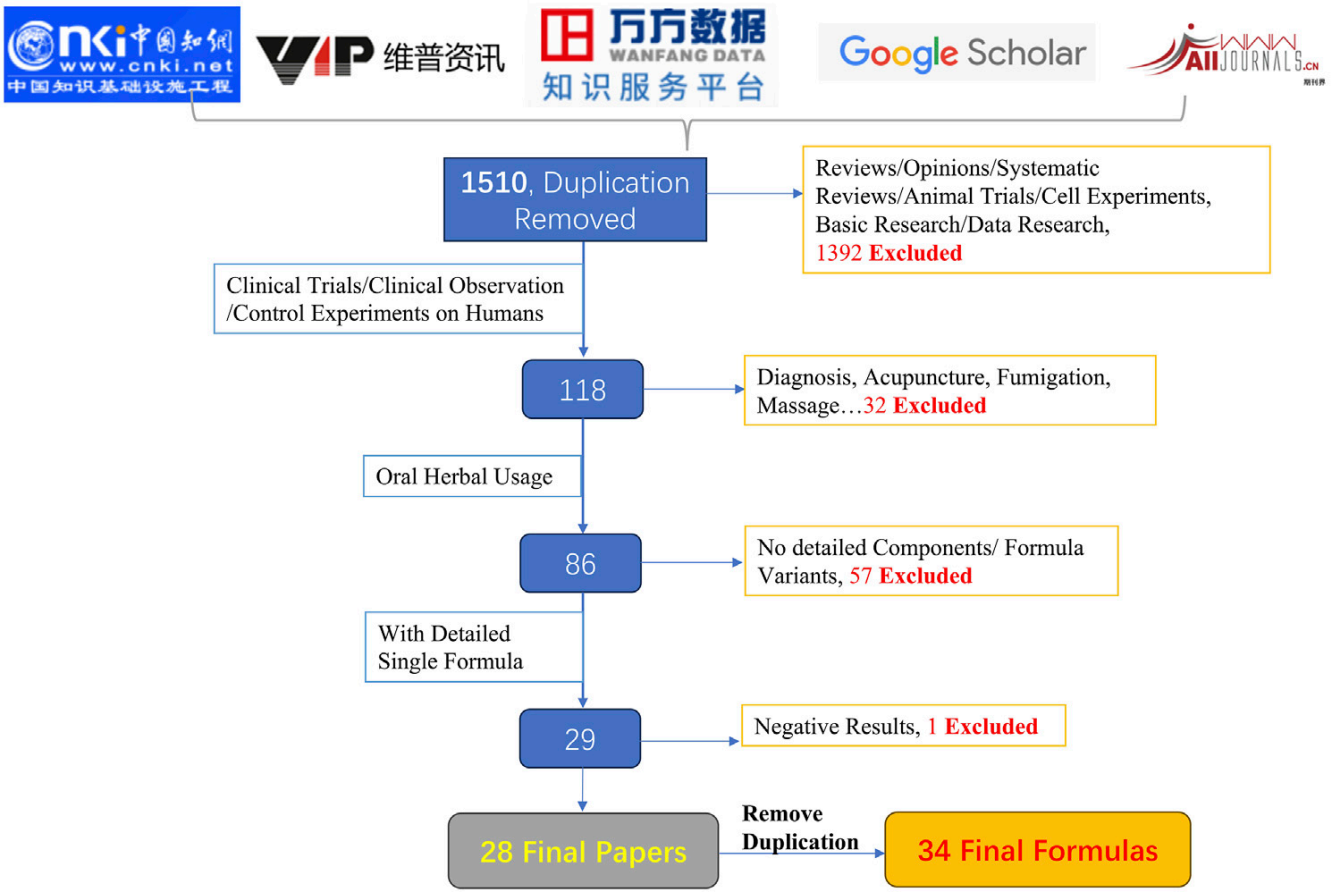
Literature screening flowchart. Flowchart summarizing the selection criteria of clinical TCM studies across five major databases. A total of 1,510 records were reviewed, with stepwise exclusion based on relevance, intervention type, and outcome availability, resulting in 34 eligible oral herbal formulas for bipolar disorder.

The selection of formulas was achieved through the literature review. The selection criteria are shown in Figure 1. To align with the study’s objective of evaluating the efficacy of orally administered TCM botanical drugs in treating bipolar disorder, we included only controlled clinical studies reporting positive outcomes. Studies involving non-oral interventions (e.g., acupuncture, fumigation, TCM-based psychotherapy), reviews, data-mining analyses, preclinical research (e.g., cell or animal studies), and trials with negative outcomes were excluded. According to the selection criteria, a total of 34 formulas were selected. According to the Pharmacopoeia of the People’s Republic of China 2020 Edition (Pharmacopoeia) (Xu et al., 2021), all the formula metabolites were standardized into standard Latin names.

### 2.3 Formulas pattern analysis

Using Python, we analyzed the appearance frequency of each botanical drug and the distribution of the number of botanical drugs in different botanical drug formulas. The botanical drugs with top 20 frequency were selected for hierarchical cluster analysis. To reveal the botanical drugs co-occurrence regularities, the *Apriori* algorithm was used for association analysis on these botanical drugs. Core botanical drugs were screened for further analysis. Hierarchical clustering was used to identify groups of frequently co-prescribed botanical drugs, revealing common prescription patterns in TCM practice. The *Apriori* algorithm further uncovered statistically significant herb pairings, highlighting potential synergistic relationships within treatment strategies. The combination of these two methods revealed the statistical co-occurrence patterns of commonly used botanical drugs and helped identify potential synergistic relationships within actual formulas, thereby enabling the selection of the most representative core herbal medicines for BD treatment.

### 2.4 Bioinformatics targets screening for BD

Two gene-disease association databases, namely, DisGeNET (Pinero et al., 2020) and GenCards (Stelzer et al., 2016), were utilized for BD targets screening. The standardized terms for BD were defined by Medical Subject Heading (MeSH) (Baumann, 2016), specific search terms are detailed in Supplementary Table S1. These terms were sequentially entered into both databases to retrieve associated genes and proteins. Within each database, the results obtained from different search terms were combined by taking the union of all returned gene/protein sets. The intersecting results from these two databases were identified as the gene/protein targets related to BD (TRB).

### 2.5 Bioinformatics functional analysis for core botanical drugs

To further understand the biological mechanism of these core botanical drugs in treating BD, we retrieved the botanical drug metabolites and the targets of these metabolites (TOCs) via BATMAN-TCM 2.0 database (Kong et al., 2024), for each core botanical drug, respectively. The intersection of the TRB and the TOC was taken for the target that these botanical drugs can affect, as the druggable targets. Next, Gene Ontology (GO) and Kyoto Encyclopedia of Genes and Genomes (KEGG) pathway enrichment analyses were conducted on these druggable targets.

### 2.6 Network pharmacology analysis for core botanical drugs

To pinpoint the core interaction metabolites and druggable targets from these core botanical drugs, as well as to reveal the multi-target effects, we conducted network pharmacology analysis for the core botanical drugs. A comprehensive botanical drugs-metabolites-targets network was established, with the key metabolites and key targets highlighted. All network visualization in our work was conducted using Cytoscape 3.10.2.

### 2.7 Cell culture

SH-SY5Y cells (a human neuroblastoma cell line) were maintained in DMEM (4.5 g/L glucose) supplemented with 10% FBS, 1% P/S, and 1% L-glutamine. Cells were cultured at 37°C in a humidified 5% CO_2_ incubator, with the medium refreshed every 2–3 days. Cells were passaged using 0.25% trypsin-EDTA at a split ratio of 1:3–1:5.

### 2.8 MTT cell viability assay

SH-SY5Y cells were seeded in 96-well plates at 1 × 10^4^ cells/well and allowed to adhere for 24 h. Cells were treated with PA (0, 10, 20, 40, 80, 100 μM) or SA (0, 10, 20, 40, 80 μM) for 24 h. 10 μL MTT (5 mg/mL) was added to each well, followed by incubation for 4 h (protected from light). The medium was aspirated, and 100 μL DMSO was added to dissolve formazan crystals. Plates were gently shaken for 10 min. Absorbance was measured at 570 nm using a microplate reader. Cell viability was calculated relative to the control group (set as 100%).

### 2.9 qRT-PCR analysis

Total RNA was isolated using TRIzol reagent for RNA Extraction; 1 μg RNA was reverse-transcribed into cDNA using a High-Capacity cDNA Reverse Transcription Kit; SYBR Green Master Mix was used with the following primers: GABRA1: F: CCA​AGT​CTC​CTT​CTG​GCT​CAA​C, R: AAG​CCA​CCT​TCG​GGA​GGG​AAT​T; ESR1: F: GCT​TAC​TGA​CCA​ACC​TGG​CAG​A, R: GGA​TCT​CTA​GCC​AGG​CAC​ATT​C; β-actin (internal control): F: CAC​CAT​TGG​CAA​TGA​GCG​GTT​C, R: AGG​TCT​TTG​CGG​ATG​TCC​ACG​T under the Cycling conditions of 95°C for 10 min (pre-denaturation), followed by 40 cycles of 95°C for 15 s and 60°C for 1 min; Data were analyzed using the 2^(-ΔΔCt)^ method.

### 2.10 Statistical analysis

Botanical drug frequency analysis was conducted by counting the number of times each botanical drug appeared in the selected formulas using Python’s Pandas and Counter modules. For hierarchical clustering, we used the SciPy library with Ward linkage and Euclidean distance. A heatmap was generated using Seaborn to visualize botanical drug co-appearance patterns. For the *apriori* algorithm in association analysis, we used Mlxtend Python module to conduct. A threshold of support ≥15%, confidence ≥80%, lift ≥1.2 was set to identify meaningful botanical drug associations. For the database screening, in GeneCards, only targets with the top 1,000 highest association scores were selected; in DisGeNET, a threshold of gene-disease association score >0.5 was applied. Botanical drug-target prediction from BATMAN-TCM was performed with a score cutoff set to ≥0.84 and an adjusted p-value <0.05. GO and KEGG enrichment analyses were performed using the clusterProfiler R package, terms with adjusted p-values <0.05 were considered significantly enriched. For experiments, all data are presented as mean ± SEM. Statistical analysis of three or more groups was performed by one-way ANOVA and Dunnett’s multiple comparisons using GraphPad Prism version 10.0 software (United States). Values of p < 0.05 were considered statistically significant.

## 3 Result

### 3.1 Statistical & pattern analysis results of formulas

**FIGURE 2:**
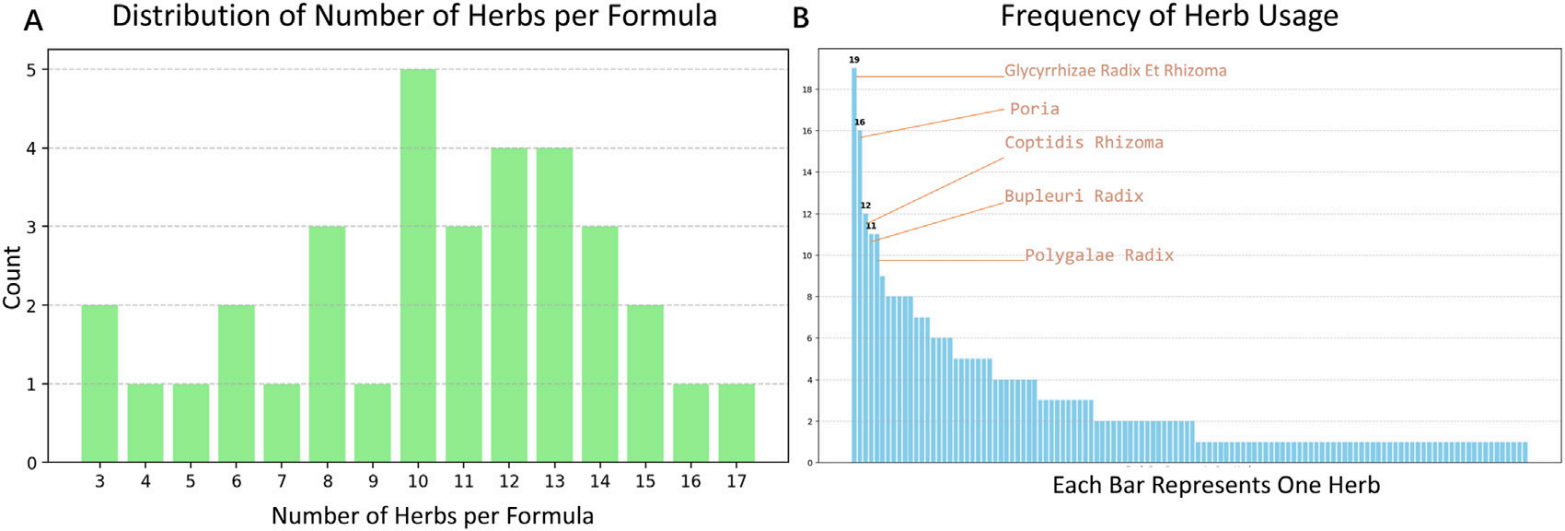
Statistics of herb composition. **(A)** Distribution of the number of herbs per formula. Most formulas contain 10–13 herbs, with the highest count being 17. **(B)** Frequency of herb usage across formulas. Glycyrrhizae Radix Et Rhizoma, Poria, Coptidis Rhizoma, Bupleuri Radix and Polygalae Radix were among the most commonly used herbs, indicating their potential central role in TCM treatment for BD.

**FIGURE 3:**
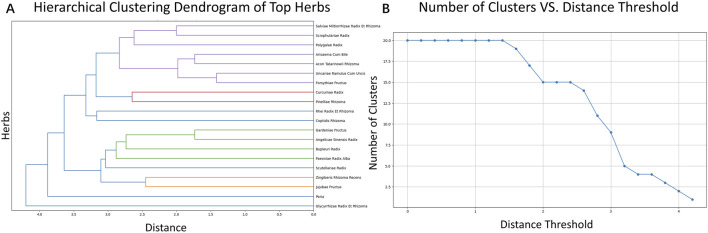
Clustering analysis of frequently used herbs. **(A)** Hierarchical clustering dendrogram based on co-occurrence patterns among the top 20 botanical drugs in formulas. Clusters are color-coded, with clear grouping patterns observed, including tight clusters between Glycyrrhizae Radix Et Rhizoma and Poria, and between Polygalae Radix and Acori Tatarinowii Rhizoma. **(B)** Cluster number changes with different distance thresholds. When the distance threshold is set to 2.5, five distinct clusters are observed: Cluster 1: Glycyrrhizae Radix Et Rhizoma & Poria; Cluster 2: Bupleuri Radix, Gardeniae Fructus & Coptidis Rhizoma; Cluster 3: Rhei Radix Et Rhizoma & Angelicae Sinensis Radix; Cluster 4: Acori Tatarinowii Rhizoma & Polygalae Radix; Cluster 5: remaining herbs including Scutellariae Radix, Paeoniae Radix Alba, and others.

The distribution of the number of botanical drugs per formula is shown in Figure 2A, with the top three frequencies highlighted. The botanical drugs with the top five usage frequencies are Glycyrrhizae Radix Et Rhizoma, Poria, Coptidis Rhizoma, Bupleuri Radix, and Polygalae Radix. The distribution of the number of botanical drugs per formula is shown in Figure 2B. In hierarchical clustering, the detailed clustering process and the selection of the distance threshold are shown in Figures 3A,B.

**FIGURE 4:**
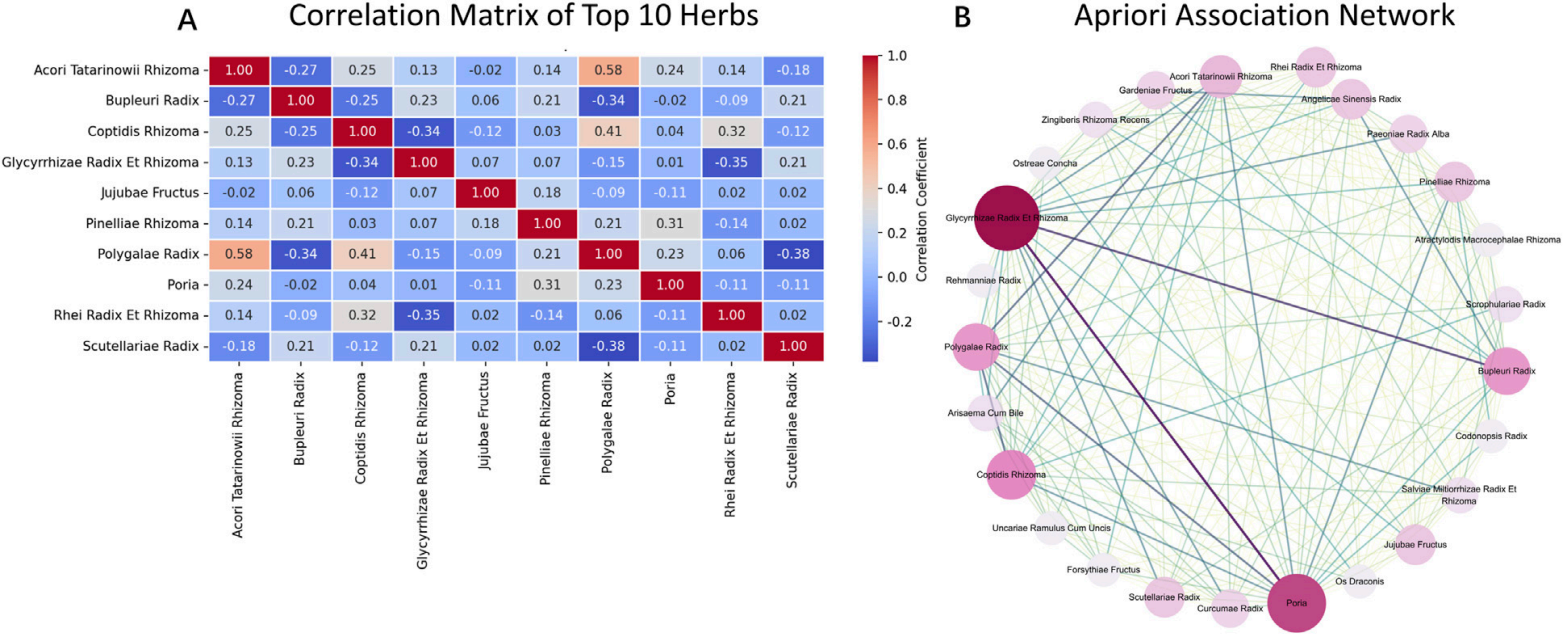
Correlation and association of key herbs. **(A)** Correlation matrix showing pairwise co-occurrence relationships among the top 10 frequently used herbs. Most pairs exhibit weak or negative correlations, while some like Acori Tatarinowii Rhizoma and Polygalae Radix show strong positive association. **(B)** Apriori association network reveals statistically significant herb-pair rules, highlighting core herbs such as Glycyrrhizae Radix Et Rhizoma and Poria as major hubs in formula design.

For the botanical drugs association, an association heatmap for the top 10 botanical drugs is shown in Figure 4A. Most botanical drugs exhibit a slight negative correlation in their co-occurrence, which may reflect some form of substitution effect. However, combinations like *Acori Tatarinowii Rhizoma* and *Polygalae Radix* show a strong positive correlation, forming a paired botanical drugs synergy. To further explore the formulas’ regularity and to identify the core botanical drugs, we conducted an *Apriori* association analysis. Figure 4B is the visualization results of the *Apriori* association analysis.

In this network, five nodes stand in the core position, namely, Glycyrrhizae Radix Et Rhizoma, Poria, Coptidis Rhizoma, Bupleuri Radix, and Polygalae Radix, which are also the top five frequency botanical drugs identified before. The tight interaction and the high frequency of these five botanical drugs form an absolute core.

### 3.2 BD druggable targets identification

The intersection of targets in DisGeNET and GeneCards forms the TRB. After overlapping the TRB and TOC, the number of druggable targets is 140.

### 3.3 Functional enrichments of druggable targets

**FIGURE 5:**
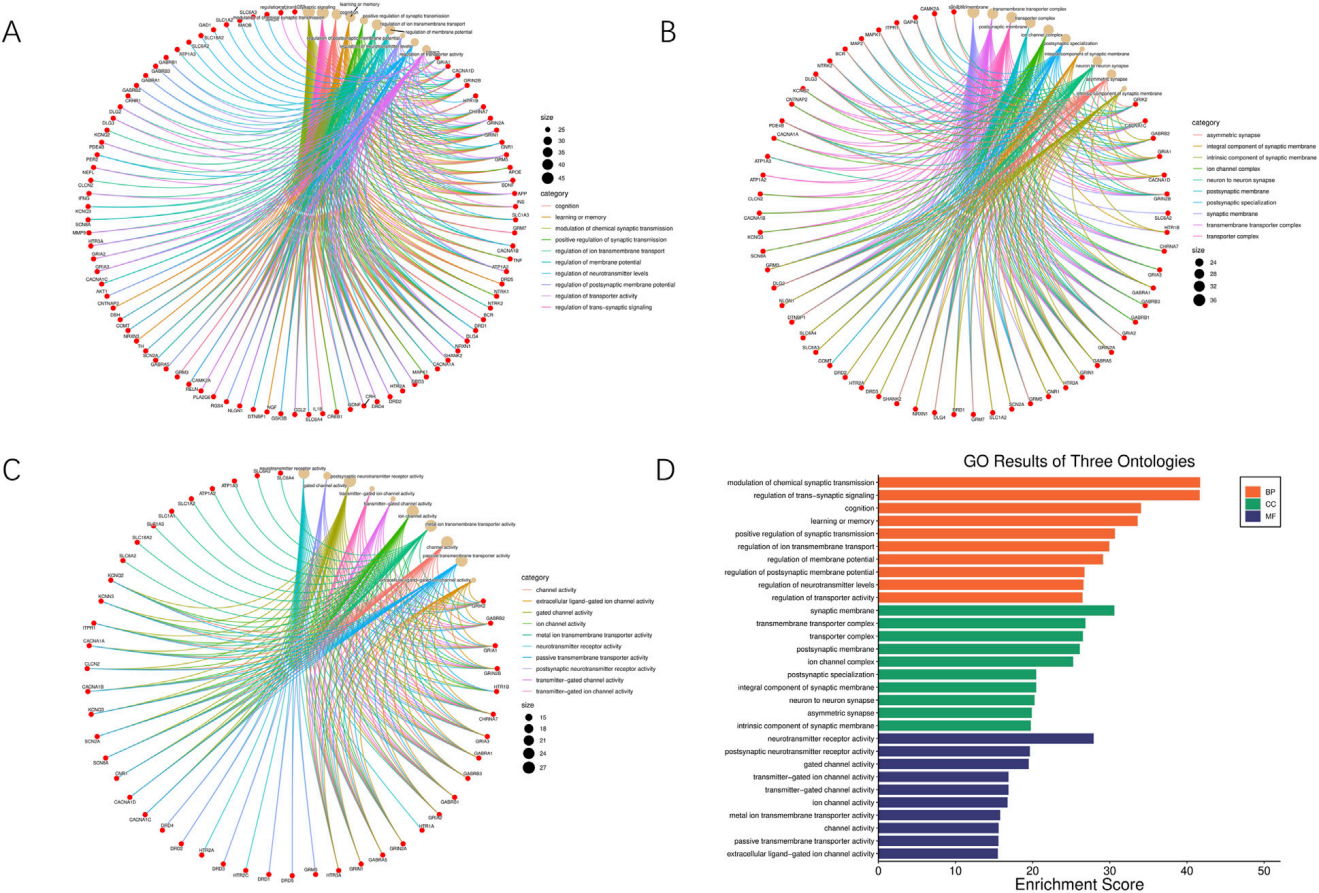
Gene Ontology (GO) enrichment analysis. **(A–C)** Network plots of enriched GO terms among the druggable targets, classified into biological processes, cellular components, and molecular functions. **(D)** Bar graph showing the top enriched GO terms across the three categories, including terms related to synaptic transmission, cognition, and membrane potential regulation.

**FIGURE 6:**
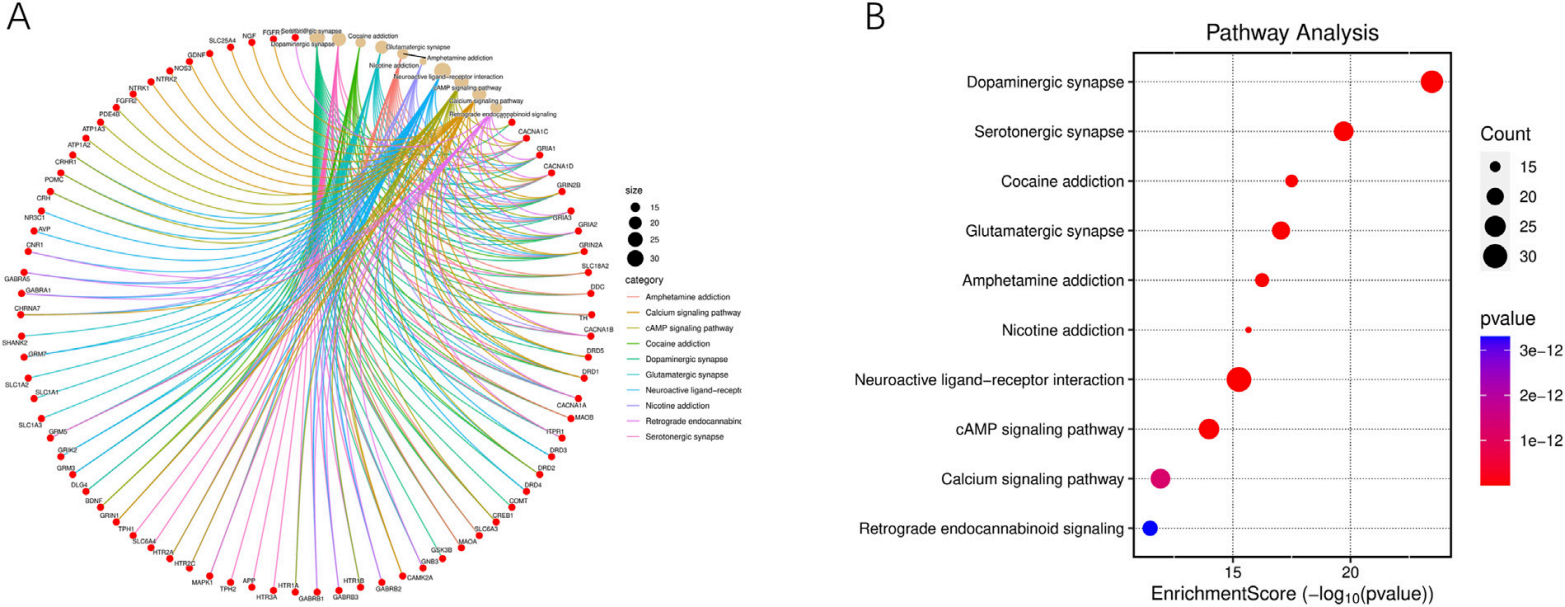
KEGG pathway enrichment analysis of druggable targets. **(A)** Chord diagram showing core KEGG pathways and associated genes involved in BD-related neural processes. **(B)** Bubble chart of enriched pathways, with bubble size reflecting gene count and color representing p-value significance.

The GO and KEGG enrichment results are shown in Figures 5, 6. The GO enrichment results indicate that these druggable targets are mainly responsible for the mechanism of signal transmission and regulation between neurons in the brain, particularly related to functions such as learning, memory, and cognition. The KEGG pathway enrichment result suggests that these targets are related to various neurotransmitter systems and signaling mechanisms in the brain, focusing on critical functions like emotion regulation, learning, memory, and especially, addiction-related pathways. This reveals how the active metabolites in these five core botanical drugs alter brain function, which is also the primary mechanism behind the therapeutic effects of these botanical drugs in treating BD.

### 3.4 Comprehensive network analysis results

**FIGURE 7:**
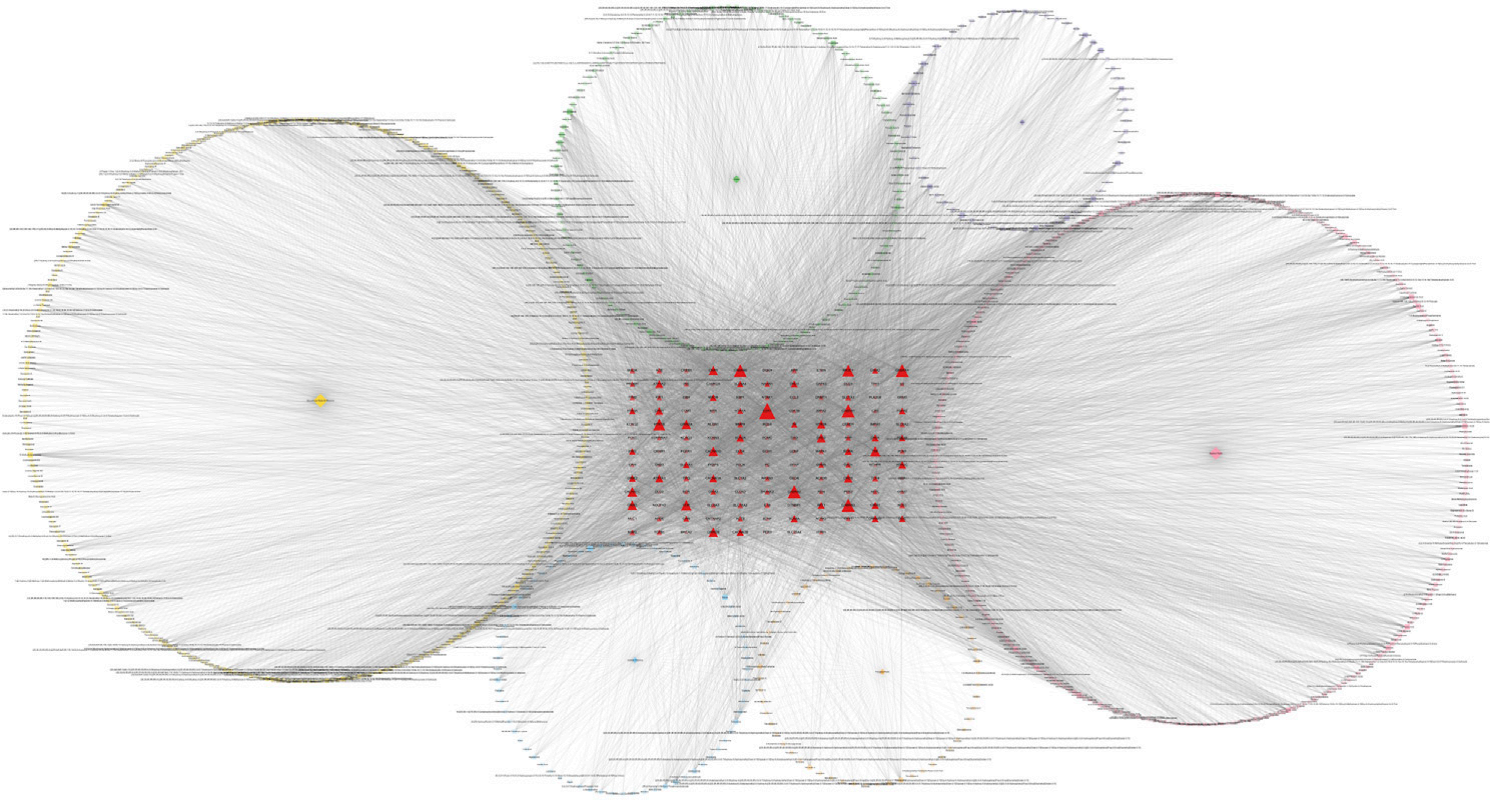
Network pharmacology map of core botanical drugs, their active metabolites, and related targets in BD. This network map visualizes the interactions between five core botanical drugs (represented as yellow pentagons), their active metabolites (shown as circles with distinct colors), and BD-related druggable targets (depicted as red triangles). The multi-origin metabolites, derived from multiple herbs, are highlighted by larger-sized circles positioned centrally. Red triangle targets vary in size, with larger nodes indicating higher degree centrality, suggesting potential therapeutic significance. Core targets such as GABRA1, GABRB2, and ESR1 are prominently positioned and densely connected, while major bioactive compounds including palmitic acid, stearic acid, betulin, and histamine exhibit broad interactions across multiple pathways. The overall topology reveals a multi-level regulation pattern, reflecting the complex, multi-target pharmacological mechanisms of TCM formulas in the treatment of BD.

Network pharmacology analysis indicates that among the numerous metabolites, the most widely acting and core metabolites are Histamine, Palmitic Acid, Stearic Acid, and Betulin. The most critical druggable targets are ESR1, GABRB3, GABRB1, GABRB2, GABRA5, and GABRA1. A large number of multi-origin metabolites demonstrate the synergistic effect of TCM formulas. The detailed network is shown in Figure 7.

### 3.5 Effects of PA and SA on SH-SY5Y cell viability

**FIGURE 8:**
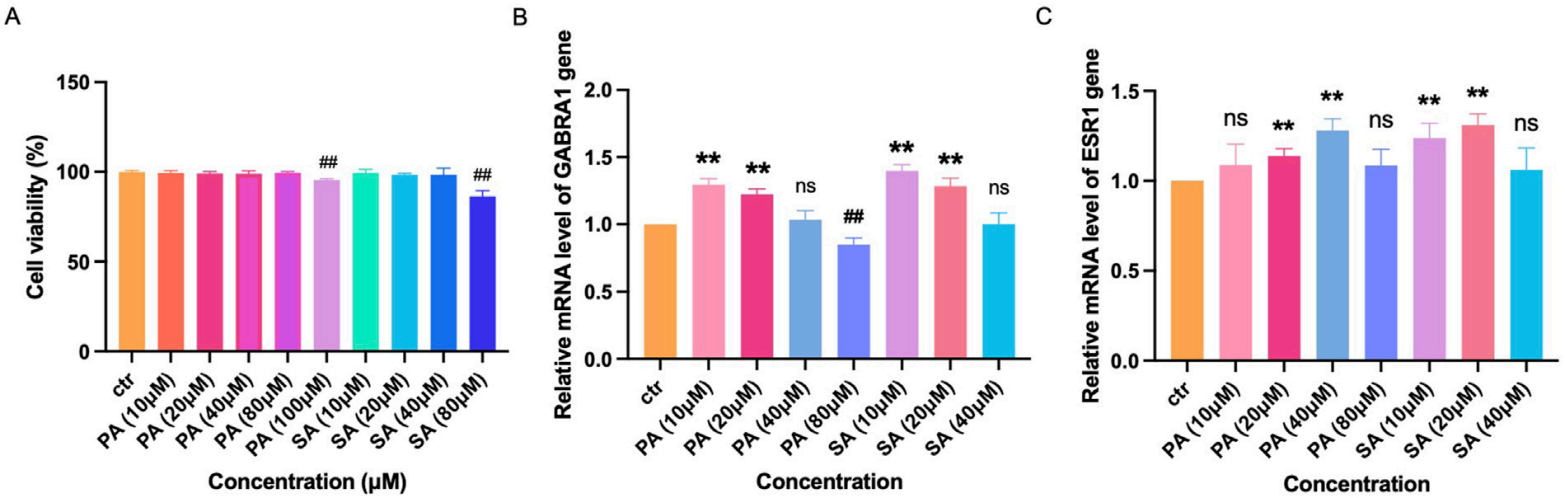
Effects of PA and SA on SH-SY5Y cells. **(A)** MTT assay showing PA and SA do not significantly reduce SH-SY5Y cell viability except at high doses (PA 100 μM, SA 80 μM). **(B)** Relative mRNA levels of GABRA1, showing bidirectional regulation by PA (up at low dose, down at high dose) and consistent upregulation by SA. **(C)** ESR1 expression is significantly upregulated by both PA and SA at moderate doses, indicating possible receptor-mediated neuroregulatory effects. ## indicates a statistically significant downregulation compared to control group, while ∗∗ indicates a significant upregulation.

MTT assay revealed that PA (10, 20, 40, 80 μM) and SA (10, 20, 40 μM) exhibited no effects on SH-SY5Y cell viability (Figure 8A). Meanwhile, PA at 100 μM significantly reduced cell viability, and SA at 80 μM showed the inhibition (p < 0.01, Figure 8A).

### 3.6 PA and SA regulate GABRA1 and ESR1 expression

qRT-PCR results demonstrated that PA shows a bi-regulation to the expression of GABRA1 gene, where low dose (10, 20 μM) can upregulate the expression and high dose (80 μM) downregulated GABRA1 expression (p < 0.01, Figure 8B) and upregulated ESR1 (20, 40 μM) (p < 0.01, Figure 8C). Conversely, SA (10, 20 μM) significantly increased both GABRA1 expression (p < 0.01, Figure 8B) and ESR1 levels (10, 20 μM), compared to the control group (p < 0.01, Figure 8C).

## 4 Discussion

TCM offers extensive clinical experience, but this knowledge often struggles to translate into drug development insights due to non-standardized approaches in syndrome differentiation and treatment. Traditional methodologies contribute to redundancy and noise in clinical data. Variations in formulas among TCM pharmacists, influenced by personal experience and clinical style, further complicate this issue (Zhuang et al., 2021). TCM have potential advantages in overall regulation of BD, such as improving sleep, mood swings, and reducing metabolic side effects. They are particularly suitable for assisting in relieving the side effects of standard drugs such as lithium salts and antipsychotics (such as insomnia and cognitive impairment), but they are slow to take effect and lack standardization. Although standard drug therapy can quickly control acute attacks and reduce the risk of suicide, it faces long-term side effects, including renal damage, diabetes insipidus and lithium poisoning, and weight gain. In the future, multicenter RCTs are needed to verify the synergistic effect of combined TCM and Western medicine treatment, prolong the remission period, and use omics technology to analyze the multi-target mechanism of TCM, promote the integration of evidence-based medicine and traditional medicine, and achieve individualized stratified treatment. Although there is some research on TCM treatment for BD, there has been a lack of systematic data-mining studies involving multiple clinical projects. Our research fills this gap. We employed various statistical methods and machine learning algorithms to perform a DMDA holistic analysis, uncovering patterns in TCM botanical drug usage for BD treatment. Our study utilized data from hundreds of clinically effective cases, significantly enhancing the reliability of the findings. This highlights the strength of DMDA in using large datasets to minimize the randomness or biases of individual experiments.

Our study identified 34 different formulas for BD over the past decade. However, a common pattern emerged through our DMDA analysis: the consistent use of five core botanical drugs. Frequency analysis showed significant enrichment of these botanical drugs, and clustering analysis distinctly separated them from others. Association analysis highlighted their central role in the formula network and their interconnections. These five core botanical drugs could be pivotal in extracting active metabolites and developing new drugs in the future.

The five core botanical drugs identified in this study—Glycyrrhizae Radix Et Rhizoma, Poria, Coptidis Rhizoma, Bupleuri Radix, and Polygalae Radix—reflect the traditional TCM principles of syndrome differentiation and botanical drug-pattern correspondence. In TCM theory, the pathogenesis of BD is primarily attributed to disharmony between the heart and liver, phlegm-fire disturbing the mind, imbalance of Yin and Yang, and unrest of the Shen (spirit). Common TCM syndromes include Liver Qi stagnation, phlegm-fire disturbing the heart, heart-spleen deficiency, Yin deficiency with internal heat, and heart-kidney disharmony. Bupleuri Radix is effective in soothing liver Qi and treating emotional depression (Zhong et al., 2024); Coptidis Rhizoma clears fire and calms the mind, applicable for manic agitation caused by phlegm-fire and Yin deficiency heat (Wang et al., 2018); Poria strengthens the spleen, eliminates dampness, and calms the Shen, often used for cognitive decline and emotional instability (Hao et al., 2021); Polygalae Radix nourishes the heart and kidney while opening the orifices, useful in cognitive and emotional symptoms related to Heart-kidney disharmony (Liu et al., 2021); and Glycyrrhizae Radix harmonizes Qi, alleviates tension, and enhances the synergistic effects of other botanical drugs (Li et al., 2019). These botanical drugs address key organ systems (heart, liver, spleen, and kidney) and exert multi-dimensional therapeutic effects, including tranquilization, emotion regulation, heat-clearing, and phlegm transformation. Together, these botanical drugs address key organ systems (heart, liver, spleen, kidney) with tranquilizing, emotion-regulating, heat-clearing, and phlegm-transforming effects. Notably, our study employs DMDA rather than strictly adhering to traditional TCM frameworks. Therefore, botanical drugs like Glycyrrhizae Radix Et Rhizoma and Poria, although traditionally viewed as harmonizing or supportive rather than directly therapeutic for emotional disorders, were thus retained to accurately reflect realist botanical drugs formula patterns.

The use of databases to identify disease-associated targets and metabolites for drug development is a well-established technique, but our DMDA pipeline addresses several limitations found in current bioinformatics approaches. This comprehensive approach allowed us to identify high-confidence disease targets, establishing a solid foundation for further research. For example, in the analysis of BD-related genes, we identified key modules and hub genes. The significance of these genes has been corroborated by previous studies. Additionally, we provided clinical insights for drug development. For instance, there has been controversy regarding the role of GABRB2 in mood stabilization (Gao et al., 2022). Initial studies suggested that GABRB2 does not play a significant role, while later research indicated its importance, showing that metabolites like Valproic Acid can mitigate BD symptoms through GABRB2-related pathways (Chen et al., 2009). Our DMDA analysis, leveraging extensive data, confirms the crucial role of GABRB2 in BD pathogenesis.

To further validate the bioinformatics predictions, we designed *in vitro* experiments using SH-SY5Y neuroblastoma cells, a model system for studying neuronal function and drug responses. PA and SA were selected based on their high interaction scores with core targets (GABRA1 and ESR1) in network pharmacology analysis. Our dose-response experiments, which aimed to mimic physiological concentrations, revealed that PA and SA can bidirectionally regulate GABRA1 and ESR1 depending on the dosage. This bidirectional regulation is particularly relevant for the treatment of bipolar disorder, where modulation of GABA activity is crucial. At lower doses, PA and SA may enhance GABA activity, providing calming effects, while higher doses may suppress excessive GABA activity to prevent mood depression. These findings underscore the importance of dosage optimization in TCM-based formulas and align with the network-predicted synergistic effects of botanical drug metabolites. They also provide mechanistic insights into how core botanical drugs modulate key neuropsychiatric targets, offering a nuanced approach to managing bipolar disorder.

This study also has some limitations. Our DMDA is based on clinical cases recorded in Chinese literature. Although TCM has a long and extensive history of application in Asia, there are still concerns about its applicability in different populations. Our study used the DisGeNET and GeneCards databases to identify disease-associated genes. Although both databases are comprehensive and authoritative resources, they do not provide demographic information about the origin of these genes or targets. Moreover, data mining and network analysis are highly predictive in nature, and their results should be interpreted with caution. Therefore, although this study provides validation at the cellular level, its therapeutic effects in populations with different demographic characteristics should still require careful and critical interpretation. Future studies will include animal experiments and pharmacokinetic analysis under different epigenetic conditions to fully evaluate the reliability and generalizability of the study results.

## 5 Conclusion

The treatment of BD with traditional Chinese medicine is centered around five core botanical drugs: *Glycyrrhizae Radix Et Rhizoma*, *Poria*, *Coptidis Rhizoma*, *Bupleuri Radix*, and *Polygalae Radix*. The primary mechanism behind the therapeutic effects of these botanical drugs is their interaction with the GABRA1 and ESR1.

## Data Availability

The original contributions presented in the study are included in the article/Supplementary Material, further inquiries can be directed to the corresponding authors.
