# Supplementary material for: Exploring the patterns in traditional Chinese medicine for bipolar disorder: a data-driven network approach
Source: Front Pharmacol. 2025 Jun 4;16:1524345. doi: 10.3389/fphar.2025.1524345 (PMC12174447; doi:10.3389/fphar.2025.1524345)
Supplement: Supplementary file 3 [file Table1.docx]

**Supplements**

| Database | Search Term |
| --- | --- |
| DisGeNET | ("Bipolar Disorders" OR "Bipolar I Disorder" OR "Mixed Bipolar Disorder" OR "Bipolar Affective Disorder" OR "Bipolar I disorder, hypomanic" OR "Bipolar Disorder, Type 1" OR "Bipolar II Disorder" OR "Bipolar Affective Disorder, Depressed, Unspecified Degree") |
| GeneCards | ("Bipolar Disorder" OR "Bipolar Affective Disorder" OR "Manic Depression" ) |

**Sup 1.** The search strategy in each database.
